# Proliferation Potential of Müller Glia after Retinal Damage Varies between Mouse Strains

**DOI:** 10.1371/journal.pone.0094556

**Published:** 2014-04-18

**Authors:** Akiko Suga, Kazuyo Sadamoto, Momo Fujii, Michiko Mandai, Masayo Takahashi

**Affiliations:** Laboratory for Retinal Regeneration, Center for Developmental Biology, RIKEN, Minatojima, Chu-O-ku, Kobe, Japan; Center for Regenerative Therapies Dresden, Germany

## Abstract

Retinal Müller glia can serve as a source for regeneration of damaged retinal neurons in fish, birds and mammals. However, the proliferation rate of Müller glia has been reported to be low in the mammalian retina. To overcome this problem, growth factors and morphogens have been studied as potent promoters of Müller glial proliferation, but the molecular mechanisms that limit the proliferation of Müller glia in the mammalian retina remain unknown. In the present study, we found that the degree of damage-induced Müller glia proliferation varies across mouse strains. In mouse line 129×1/SvJ (129), there was a significantly larger proliferative response compared with that observed in C57BL/6 (B6) after photoreceptor cell death. Treatment with a Glycogen synthase kinase 3 (GSK3) inhibitor enhanced the proliferation of Müller glia in 129 but not in B6 mouse retinas. We therefore focused on the different gene expression patterns during retinal degeneration between B6 and 129. Expression levels of Cyclin D1 and Nestin correlated with the degree of Müller glial proliferation. A comparison of genome-wide gene expression between B6 and 129 showed that distinct sets of genes were upregulated in the retinas after damage, including immune response genes and chromatin remodeling factors.

## Introduction

Recent evidence indicates that Müller glia could be a source of neuronal regeneration after retinal damage in mammals [1], [2], [3], [4], [5]. In rodent models, after acute damage to the neural retina by an intravitreal injection of *N*-Methyl-_D_-aspartate (NMDA) [1], [3] or due to a retinal explant being performed [2], Müller glia re-entered the cell cycle and became retinal progenitor-like cells with the expression profile of retinal progenitors. After two or more weeks, some of these cells differentiated into photoreceptors or inner retinal neurons, as indicated by their position and the expression of specific markers for retinal neurons. Although the numbers of proliferative Müller glia in the rodent models were rather small for replacing the damaged retinal neurons, the addition of mitogens, such as Wnt3a [2], a combination of Wnt3a and Jagged [4], EGF [3], a combination of insulin and FGF1 [3], or Shh [6], remarkably expanded the proliferative Müller glia, as well as the number of Müller glia-derived retinal neurons. Thus, the regeneration of retinal neurons through the stimulation of Müller glia is a possible therapeutic strategy for treating mammalian retinal degeneration.

In addition to mitogenic factors, the regulation of the initiation of Müller glial proliferation is another essential issue in considering intrinsic regeneration. The molecular mechanisms that regulate Müller glial re-entry into the cell cycle and/or the transition into retinal progenitor-like cells have been intensively studied in zebrafish models [7], [8], [9], [10]; in contrast, the use of rodent retina has been very limited, most likely due to the smaller degree of Müller glia proliferation [11]. In addition, only certain types of damage seem to cause the proliferation of Müller glia in the adult mouse retina, whereas in zebrafish, Müller glia respond to various neuronal damages, such as intense light stimulation [12], neurotoxin injection [13], retinal puncture [14], and the genetic removal of specific types of neurons [15]. In studies using adult mouse models, NMDA-induced inner nuclear cell death caused proliferation of Müller glia *in vivo* [3], but photoreceptor damage due to intense light stimulation or a genetic model of retinitis pigmentosa did not cause Müller glial proliferation, even with an intravitreal injection of EGF protein [16]. A recent report also showed that the number of proliferative Müller glia in retinal explants was dependent on the age of the mice, and BrdU incorporation of Müller glia was not detected when mice were older than three weeks of age [17]. These data suggest that there may be an inhibitory mechanism that limits the proliferation of Müller glia in the mammalian retina, even after damage.

Herein, we report that the proliferative potency of Müller glia in response to specific retinal damage was different across mouse strains: the number of BrdU-incorporating proliferative Müller glia was significantly less in retinal explants from adult C57BL/6 (B6) mice compared with those from 129×1/SvJ (129) or BDF1 mice. Furthermore, the addition of a Glycogen synthase kinase 3 (GSK3) inhibitor significantly increased the proliferative Müller glia in the retinal explants from 129 and BDF1 mice, but not in the B6 retinal explants. The transcriptional profile of genes associated with a suppressive or permissive retinal environment for the proliferation of Müller glia, as well as the expression of chromatin remodeling factors, differed between the B6 and 129 mouse retinas after damage.

## Materials and Methods

### Animals

All animal experiments were conducted with the approval of the RIKEN Center for Developmental Biology Ethical Committee (no AH18-05-23). Male C57 BL/6N and 129×1/SvJ mice were purchased from Nihon SLC, and male BDF1 mice were purchased from CLEA Japan. The animals were maintained under a 12 h light/dark cycle with access to food and water ad libitum.

### Retinal explant cultures

Retinal explant cultures were prepared as previously described with minor modifications [18]. Briefly, eyes were enucleated from mice, and the neural retina was separated from the cornea, the sclera, the lens, the iris, the ciliary body, and the pigmented epithelium. The neural retina was placed onto a microporous membrane (30 µm in diameter; Millicell-CM; Millipore) with the ganglion cell layer up in a six-well culture plate. Each well contained 1 ml of culture medium, which consisted of 50% minimum essential medium/HEPES (Sigma), 25% HBSS (Gibco) supplemented with 200 µM L-glutamine and 5.75 mg/ml glucose. Explants were maintained in a humidified atmosphere of 5% CO_2_ and 95% air at 35.5°C. The culture medium was changed every other day. 5-ethynyl-2′-deoxyuridine (EdU) (5 µg/ml, Molecular Probes) was added to the culture medium for 4 days. The GSK3 inhibitor Chir99021 (30 µM or 15 µM, Stemgent) was applied to the culture medium for the indicated time period. The control group in all sets of experiments received treatment with the corresponding vehicle (0.05% DMSO) and EdU (5 µg/ml).

### Fixation, sectioning, and immunostaining

For whole-mount immunostaining of the retinal explants, the tissues were fixed with 4% paraformaldehyde (PFA) in 1 M phosphate buffered saline (PBS) at room temperature for 1 hour, washed twice with PBS with 0.5% of Triton X-100 (Nacalai Tesque), and incubated with 0.05% of Trypsin-EDTA (Gibco) at 37°C for 10 minutes. Samples were washed twice with PBS, blocked in PBS with 5% normal goat serum at room temperature for 30 minutes, and incubated with antibodies in PBS with 0.5% Triton X-100 and 1% goat serum for 3 over nights. After washing twice with PBS with 0.05% Tween-20 (Nacalai Tesque), the samples were incubated with secondary antibodies in Real Antibody Diluent (Dako) overnight. EdU staining was performed using a Click-iT EdU Alexa Fluoro 488 imaging kit (Molecular Probes). For cryopreservation, tissues were fixed with 4% PFA in PBS, cryoprotected in 30% sucrose overnight, and then frozen in OTC (Sakura). For paraffin-embedded sections, tissues were fixed with Superfix (Kurabo) at 4°C overnight. The primary antibodies were as follows: rabbit anti-Sox9 (Millipore), mouse anti-Glutamine synthetase (Chemicon), rabbit anti-Glutamine synthetase (Sigma), mouse anti-Ki67 (BD Pharmingen), rabbit anti-Pax6 (Covance), mouse anti-Pax6 (R&D Systems), sheep anti-Chx10 (ExAlpha), rabbit anti-Cyclin D1 (Thermo Scientific), mouse anti-Cyclin D3 (Cell Signaling), and rat anti-Nestin (BD Pharmingen). The secondary antibodies were as follows: Alexa 546-conjugated goat anti-rat IgG, Alexa 488-conjugated goat anti-mouse IgG, Alexa 546-conjugated goat anti-rabbit IgG, Alexa 546-conjugated donkey anti-mouse IgG, and Alexa 488-conjugated donkey anti-sheep IgG.

Apoptotic DNA fragmentation was detected by terminal deoxynucleotidyl transferase-mediated biotinylated UT nick end labeling (TUNEL) with the *In Situ* Cell Death Detection kit (Roche). Cell nuclei were counterstained with 4′, 6-diamidino-2-phenylindole (DAPI) (Invitrogen).

### Statistical analysis

The number of EdU-positive cells within an area (320 µm×320 µm) in the central region of the flat-mounted retina (0.5 mm to 0.7 mm from the optic disc) was counted, and a total of four areas were examined for each retinal explant. The number of Cyclin D1 positive Müller glia or the number of Pax6 and Chx10 positive cells within an area (320 µm×320 µm) was counted in the individual sections. Six areas were examined for each retina. Data were pooled from three retinal explants for Cyclin D1 positive cells and two retinal explants for Pax6 and Chx10 double positive cells; data were expressed as the mean ± standard error. Confocal images were obtained using a Zeiss LSM 510 or a Zeiss LSM 700.

### RT-PCR

Total RNA was extracted with TRIzol (Invitrogen), treated with DNase, and reverse transcribed with SuperScript III First Strand synthesis System (Invitrogen) following the manufacturer's instructions. The cDNA was used as a template for each PCR experiment using ExTaq (Takara). The primer sets were as follows: *Cyclin D1* (Fw: 5′-TCTGTTCTCGCACCACCGGGA-3′, Rv: 5′-GGGGGCAGCGGCAAGAATGT-3′), *Cyclin D3* (Fw: 5′-GCGTACCCTGACACCAATCT-3′, Rv: 5′-CACAACTTCTCGGCAGTCAA-3′), Nestin (Fw: 5′-CTCGAGCAGGAAGTGGTAGG-3′, Rv: 5′-CTTGGGACCAGGGACTGTTA-3′), and *β-actin* (Fw: 5′-CGAGCGGTTCCGATGCCCTG-3′, Rv: 5′-ACGCAGCTCAGTAACAGTCCGC-Rv). PCR products were electrophoresed on a 2% agarose gel and detected under UV illumination. The intensity of each band was quantified using ImageJ software [19].

### Microarray analysis

Two retinas were used for each condition. Total RNA was extracted from the mouse retinal tissues using TRIZOL. The extracted RNA was further purified with a spin column from an RNeasy mini kit (QIAGEN). RNA was labeled with a 3′ IVT Express Kit (Affymetrix) and hybridized to a Mouse Genome 430 2.0 Array chip (Affymetrix) following the manufacturer's instructions. The labeling, hybridization, signal detection, and hierarchical clustering were performed by the CDB Gene Chip Service. To identify the transcripts with significantly changed expressions between B6 and 129 in the normal retina, in the retinal explant after three days of culture, and in the retinal explant cultured with a GSK3 inhibitor, a statistical test with the eBayes method was applied to obtain P-values of significant change for each probe set and to estimate the false discovery rate (FDR) and q-value. Probe sets with a FDR<0.05 were chosen as significant. One thousand probe sets with the most fold changes were selected to make a heatmap. Functional Annotation Clustering was performed by DAVID Bioinformatics Resources. The data were submitted to the NCBI Gene Expression Omnibus (GSE54056).

### In situ hybridization

In situ hybridization was performed as described previously. Antisense digoxigenin (DIG) labeled RNA probes were synthesized using a DIG RNA labeling mix (Roche).

## Results

### The Müller glial proliferative response to retinal damage varies across mouse strains

**Figure 1:**
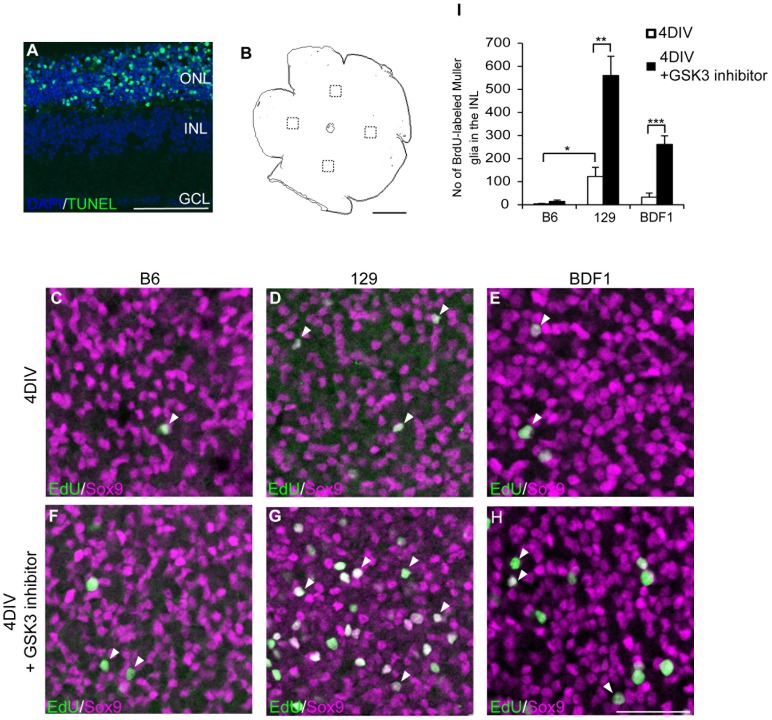
The number of proliferative Müller glia is different between mouse strains. (A) TUNEL staining of a retinal explant at 2DIV. TUNEL positive cells (green) were detected in the ONL of the retinal explant. (B) Schematic diagram of an image of the whole mount immunostaining of a retinal explant. Dotted rectangles indicate the areas where confocal images were taken. (C–H) Whole-mount immunostaining of EdU (green) and Sox9 (magenta) in the retinal explants from B6 (C, F), 129 (D, G), and BDF1 (E, H) at 4DIV, treated with vehicle (C–E), or the GSK3 inhibitor Chir99021 (F–H). Nuclei of EdU-positive, proliferating Müller glia are indicated by arrowheads. (I) Quantification of EdU-positive Müller glia in the INL at 4DIV. Open bars indicate vehicle-treated and black bars indicate Chir99021-treated retinal explants. * P<0.05, ** P<0.005, *** P<0.001. ONL: outer nuclear layer, INL: inner nuclear layer, GCL: ganglion cell layer. Scale bars: A 100 µm, B 1 mm, and C–H 50 µm.

We previously reported that retinal Müller glia proliferated and generated retinal progenitor-like cells after acute damage *in vivo* and *in vitro* using adult rat models [1], [2]. To examine whether this proliferation and de-differentiation of Müller glia also occurred in the mouse retina after damage, we used retinal explant cultures from different strains of adult mice (9 to 10 weeks old). As in the rat retina, TUNEL-positive apoptotic cells were detected in the outer nuclear layer (ONL) after 3 days of culture (3DIV) (Fig. 1A), indicating that photoreceptor cells were mainly damaged in this retinal explant culture. However, in contrast to the previous report using rat models, when the neural retina from a B6 mouse was isolated and cultured for 4 days, EdU-positive and Sox9-positive proliferative Müller glia were only scarcely detected (3.75±2.23 cell per field, mean ± standard error) in the inner nuclear layer (INL) of the central region of the retina (Fig. 1C and I). However, when we tested the retinal explants from 129 mice, the INL of the central region of the retina contained a significantly larger number of EdU-positive Müller glia (122.33±39.3 cells per filed, p = 0.021, Fig. 1D; arrowheads, and I). Because B6 mice are pigmented and 129 mice are agouti, we tested another pigmented mouse strain, BDF1. The INL of the retinal explants from the BDF1 mice contained an intermediate number of EdU-positive Müller glia (33±17.3 cells per field) at 4DIV (Fig. 1E, arrowheads, and I).

Previous reports showed that the addition of Wnt3a [2] or EGF [3] remarkably increased the number of proliferative Müller glia in the damaged rodent retina. Canonical Wnt signaling and EGF signaling are mediated by the inactivation of a GSK3 protein [87], and, indeed, a GSK3 inhibitor increased the number of proliferative Müller glia in the rat retinal explant [2]. Therefore, we also tested whether the addition of a GSK3 inhibitor increased the number of EdU-positive Müller glia in the retinal explant from adult mice. The addition of a GSK3 inhibitor into the culture medium did not significantly alter the number of EdU-positive Müller glia in the B6 retinal explants (14.25±5.6 cells per field, p = 0.12) (Fig. 1F and I), whereas the numbers of EdU-positive Müller glia were significantly increased in the retinal explants from the 129 and BDF1 mice (560±82.6 cells per field, p = 0.0019 in 129; 261.4±37.14 cells per field, p = 0.00099 in BDF1) (Fig. 1G and H; arrowheads, Fig. 1I). These data suggested that the degree of Müller glial proliferation and the response of Müller glia to a GSK3 inhibitor after retinal damage was affected by the genetic background of each mouse strain.

### Müller cells enter the cell cycle in both 129 and B6, but B6 cells did not progress to the S phase

**Figure 2:**
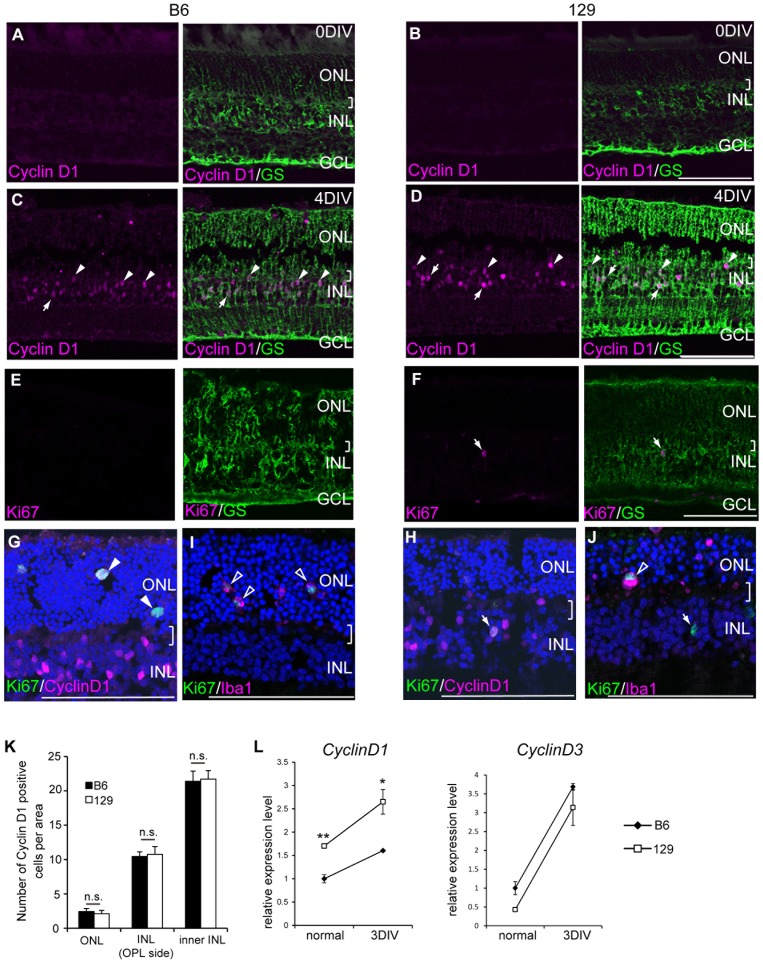
Expression of *Cyclin D1* was higher in 129 compared with B6 explants. (A–D) Immunostatining of Cyclin D1 (magenta) and GS (green) in retinal explants. Cyclin D1 was not detected in the control normal retina from B6 and 129 (A, B), but it accumulated in the nucleus of Müller glia in the retinal explants at 4DIV (arrows) (C, D). Several Cyclin D1 positive Müller glial nuclei were located in the OPL side (arrowheads). (E, F) Ki67 (magenta) was detected in the GS (green)-positive Müller glia only in the retinal explant from 129. (G, H) Cells expressing Ki67 (green) and Cyclin D1 (magenta) in a retinal explant from B6 in the ONL (arrowheads) (G) and 129 (arrow) (H). (I) Ki67 and Iba1 positive microglia in the ONL (open arrowheads). (J) Ki67 positive and Iba1 negative cells in the INL (arrow), and Ki67 positive and Iba1 positive cells in the ONL (open arrowhead). Brackets indicate OPL. Scale bar: 100 µm. (K) Number of cells positive for Cyclin D1 in the ONL and cells positive for Cyclin D1 and GS in the INL (OPL side) and the inner INL of the retinal explants. n.s.: not significant. (L) Relative expression levels of *Cyclin D1* and *Cyclin D3* transcripts in the retinal explants from B6 (black rectangle) and 129 (white square) at indicated time points.

Because few Müller glia incorporated EdU in the retinal explants from the B6 mice, we examined how Müller cells enter and progress in the cell cycle after retinal damage by immunostaining Cyclin D1 and Ki67. D-type cyclins are cell cycle markers for G1. Cyclin D1 is a major D-type cyclin expressed in embryonic retinal progenitor cells, and it is diminished after birth, which corresponds with the decrease in retinal progenitor cells [20]. As expected, Cyclin D1 protein was not detected in the adult normal retinas from B6 and 129 mice (Fig. 2A and B). However, after retinal damage, Cyclin D1 protein was detected in the nucleus of GS-positive Müller glia in both the B6 and 129 retinas, indicating that the cells entered the cell cycle in both strains (Fig. 2C and D). We noted that some Cyclin D1 positive Müller glial nuclei were located at the margin of INL and the outer plexiform layer (OPL) (Fig. 2D and E, arrowheads), which implies that these cells might be migrating to the ONL as found in the proliferating Müller glia in rat retinal explants [2]. The distribution pattern of Cyclin D1 positive Müller glia in each of the ONL, the OPL side of the INL, and the inner INL was evaluated (Fig. 2K). The distribution was not significantly different between B6 and 129 (ONL: 14.7±1.8 cells per field in B6, 12±3.7 cells per field in 129, p = 0.48; INL (OPL side): 66.7±9.2 cells per field in B6, 61±4.2 cells per field in 129, p = 0.54; inner INL: 127.3±1.5 cells per field in B6, 123±14.6 cells per field in 129, p = 0.75), but the intensity of Cyclin D1 immunostaining was generally higher in 129. Ki67 is a marker for the late G1 to M phase and indicates proliferating cells. Ki67-positive Müller glia were detected only in the retinal explant from 129 (Fig. 2E and F), and these Ki67-positive cells were mostly Cyclin D1 positive cells in the INL (Fig. 2H, arrow). Ki67 and Cyclin D1 double-positive cells were also detected in the retinal explant from B6. These cells were stained with Iba1 (Fig. 2I, open arrowheads), which indicated proliferating microglias. We also detected some Iba1 positive proliferating microglia in the 129 mouse retinal explant (Fig. 2J, open arrowhead), as well as Ki67 positive Iba1 negative cells (Fig. 2J, arrow). These data indicated that Cyclin D1 expression increased in most of the Müller glia after retinal damage in both B6 and 129, but in the B6 retina, these cells did not progress to the S phase.

Because Cyclin D1 immunostaining was higher in the 129 retinal explants, we quantitated D-type cyclins in the retinas from both strains. Cyclin D1 is expressed in the retinal progenitors during retinal development [21], [22], but Cyclin D3 appears after birth in the INL and is maintained in the Müller glia in the adult normal retina [23]. As indicators of cell cycle entry, Cyclin D1 and Cyclin D3 have been reported to increase their expression levels after retinal damage in the rat and mouse [2], [23]. Consistent with previous reports, both Cyclin D1 and Cyclin D3 mRNA increased in the retinal explant from B6 and 129, although the relative expression levels were different (Fig. 2L). Cyclin D1 expression was higher in 129 than in B6 in the normal and damaged retinas. In contrast, Cyclin D3 expression was not significantly different between B6 and 129.

### Müller glia expressed retinal progenitor markers after retinal damage in B6 and 129

**Figure 3:**
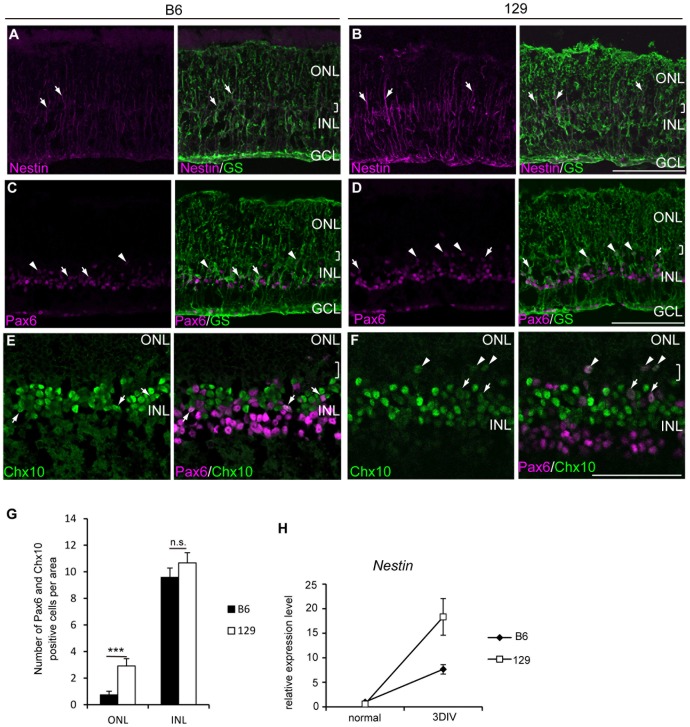
Retinal progenitor-like cells were generated in retinal explants from B6 and 129. Retinal explants from adult B6 (A, C, E,) and 129 (B, D, F,) mice were stained for indicated markers. (A, B) A portion of GS-positive Müller glia (green) expressed Nestin (magenta) in the retinal explants from B6 and 129 (arrows) at 4DIV. (C, D) Pax6 (magenta) was detected in the GS-positive Müller glia (green) (arrows) in the retinal explants from B6 and 129 at 4DIV. A number of Pax6-positive Müller glia were located in the ONL (arrowheads). (E, F) Cells in the INL co-expressed Pax6 (magenta) and Chx10 (green, arrows) in the retinal explants from B6 and 129 at 4DIV. An example of double-positive cells located in the ONL (arrowheads). (G) The number of Pax6 and Chx10 positive cells in the ONL and the INL. ***: p<0.005, n.s.: not significant. (H) Relative expression levels of *Nestin* transcripts at indicated time points. Brackets indicate OPL. Scale bar: 100 µm.

Previous studies showed that proliferating Müller glia in the damaged retina expressed retinal progenitor markers, which indicated these cells had de-differentiated [2], [3], [24]. We tested whether the de-differentiation of Müller glia also depended on the mouse strains by evaluating the expression of the retinal progenitor markers. In the normal retina from adult B6 and 129, the neural progenitor marker Nestin was only detected in the peripheral region of the retina by immunostaining (data not shown). In the retinal explants at 3DIV, some Müller glial processes were positive for Nestin in 129 (Fig. 3B), and a relatively faint signal was detected in B6 (Fig. 3A). A large increase in Nestin gene expression in the 129 retina was also confirmed by RT-PCR (Fig. 3I). We then examined the retinal progenitor markers Pax6 and Chx10. In the adult normal retina, Pax6 and Chx10 showed a distinct expression pattern, with the former expressed in the amacrine and retinal ganglion cells, and the latter expressed in the retinal bipolar cells. The coexpression of Pax6 and Chx10 is specific for retinal progenitor cells during retinal development [25]. Several GS-positive Müller glia expressed Pax6 in both B6 and 129 retinal explants (Fig. 3C and D, arrows). Similar to the Cyclin D1-positive Müller glial nuclei, some Pax6-positive Müller glial nuclei were detected near the margin of the OPL and the INL, which indicates a possible migration of these cells. We also detected a few cells that expressed both Pax6 and Chx10 in the INL of the retinal explants from B6 and 129 (Fig. 3E and F). We counted the number of Pax6 and Chx10 double-positive (Pax6+/Chx10+) cells in the ONL and the INL of the retinal explants (Fig. 3G). The majority of Pax6+/Chx10+ cells were located in the INL in both B6 and 129 (9.58±0.7 cells per area in B6; 10.67±0.76 cells per area in 129, p = 0.29); however, we detected a significantly larger number of Pax6+/Chx10+ cells in the ONL of 129 retinal explants compared to B6 (0.75±0.26 cells per area in B6; 2.92±0.55 cells per area in 129, p = 0.002). The number of BrdU-positive Müller glia in the INL in the same area of retinal sections (1.14±0.7 cells per area in B6; 3.88±0.79 cells per area in 129) was smaller than the Pax6+/Chx10+ cells in the INL, indicating that de-differentiated Müller glia did not necessarily go through division.

### Gene expression profiles of the B6 and 129 mouse retinas after damage

**Figure 4:**
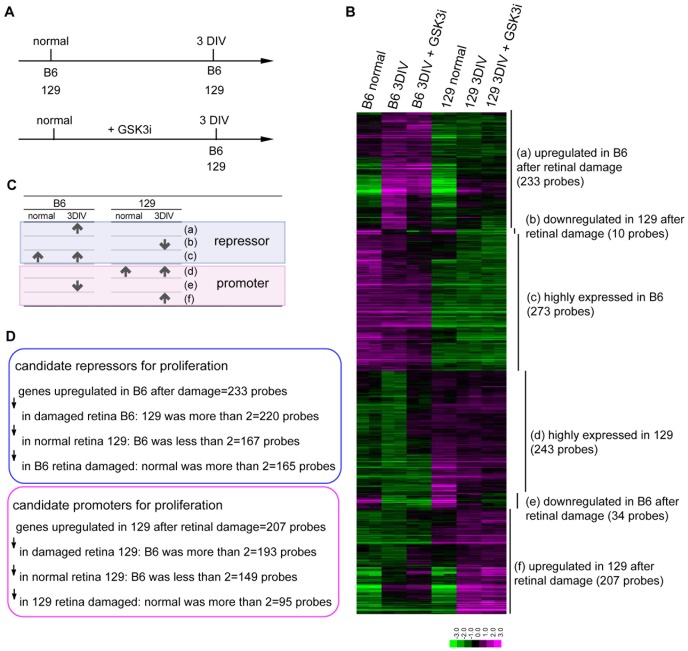
Gene expression profiles comparing normal retinas and retinal explants from B6 and 129. (A) Schematic diagram of the preparation of the retinal samples. Two retinas were prepared for each condition (1 to 6). (B) Expression profile of the 1000 most differentially expressed probe sets between the retinal explants from B6 and 129 at 3DIV. (C) Schematic diagram of the classification of the genes selected in (B). (D) Strategies to narrow down the candidate proliferation promoters and repressors.

To analyze the molecular background that contributed to the different degrees of limitation on the Müller glial proliferation, we compared the gene expression patterns in the retina between B6 and 129 after damage. Because Ki67-positive proliferating Müller glia were not detected before 3DIV, we hypothesized that the molecules contributing to a suppressive environment (B6) or a permissive environment (129) for Müller glial proliferation accumulated at 3DIV in the retinal explants. To distinguish the innate differences in the gene expression levels from the differences that appeared after retinal damage, we compared the normal retina and retinal explants at 3DIV for B6 and 129 (Fig. 4A). We selected 1000 probes that showed the largest differences between the retinal explants from B6 and 129 at 3DIV. The probes were classified into six groups (Fig. 4B, C): a) genes that were upregulated in the B6 retina after damage (233 probes), b) genes that were downregulated in the 129 retina after damage (10 probes), c) genes that were consistently highly expressed in the B6 retina (273 probes), d) genes that were consistently highly expressed in the 129 retina (243 probes), e) genes that were downregulated in the B6 retina after damage (34 probes), and f) genes that were upregulated in the 129 retina after damage (207 probes). Genes in groups (a) to (c) were expected to include candidate proliferation repressors for Müller glial proliferation, and genes in groups (d) to (f) were expected to include candidate proliferation promoters for Müller glial proliferation (Fig. 4C). To identify the characteristic functions of the genes in each group, we used Functional Annotation Clustering of the DAVID functional annotation tool [26] based on the GO terms of biological properties. As shown in Table 1, among the candidate genes of proliferation suppressors, the genes related to immune response were most highly enriched in the B6 mouse retina after damage (group (a), enrichment score (ES): 4.43), followed by the clusters “regulation of cell death”, “chemotaxis”, and “ T cell activation” (ES: 3.8, 3.6, and 2.6, respectively). Group (b) was very small with only 9 genes, and it did not make a cluster of functionally related GO terms, although 6 of 9 genes were annotated as causal genes for retinal degeneration (*Abca4*, *Elovl2*, *Fscn2*, *Nxnl1*, *Pde6c*, and *Pde6h*). Among the candidate genes of proliferation promoters in group (f), the genes upregulated in the 129 mouse retina after damage contained genes related to “cell migration” and “regulation of MAP kinase activity” (ES: 2.09 and 1.61, respectively). In agreement with our finding that the addition of a GSK3 inhibitor increased the proliferating Müller glia in the 129 retinal explants, the genes related to the “mitotic cell cycle” and “DNA replication” were highly enriched in the genes with increased expression levels by the addition of a GSK3 inhibitor in the 129 mouse retinal explants (ES: 35.64 and 10.65, respectively).

**Table 1:** Results of functional annotation clustering.

	cluster	enrichment score
(a) upregulated in B6 after damage	immune response	4.43
	regulation of cell death	3.8
	chemotaxis	3.6
	T cell activation	2.6
	positive regulation of cytokine production	2.3
	positive regulation of transport	2.2
(b) downregulated in 129 after damage	no significantly enriched cluster	
(c) highly expressed in B6	glycosylation	2
	cell cycle, M phase	1.92
(d) highly expressed in 129	regulation of microtubule cytoskeleton organization	2.6
	histone acetylation	2.13
	cell cycle, M phase	1.77
	long term potentiation	1.74
	embryonic development	1.73
(e) downregulated in B6 after damage	no significantly enriched cluster	
(f) upregulated in 129 after damage	cell migration	2.09
	protein complex assembly	1.76
	positive regulation of MAP kinase activity	1.61
	positive regulation of signal transduction	1.52
	apoptosis	1.42
upregulated by the addition of GSK3 inhibitor in 129	mitotic cell cycle	35.64
	DNA replication	10.65
	chromosome condensation	5.37
	DNA repair	5.28
	chromosome segregation	3.55
	p53 signaling pathway	3.45

**Figure 5:**
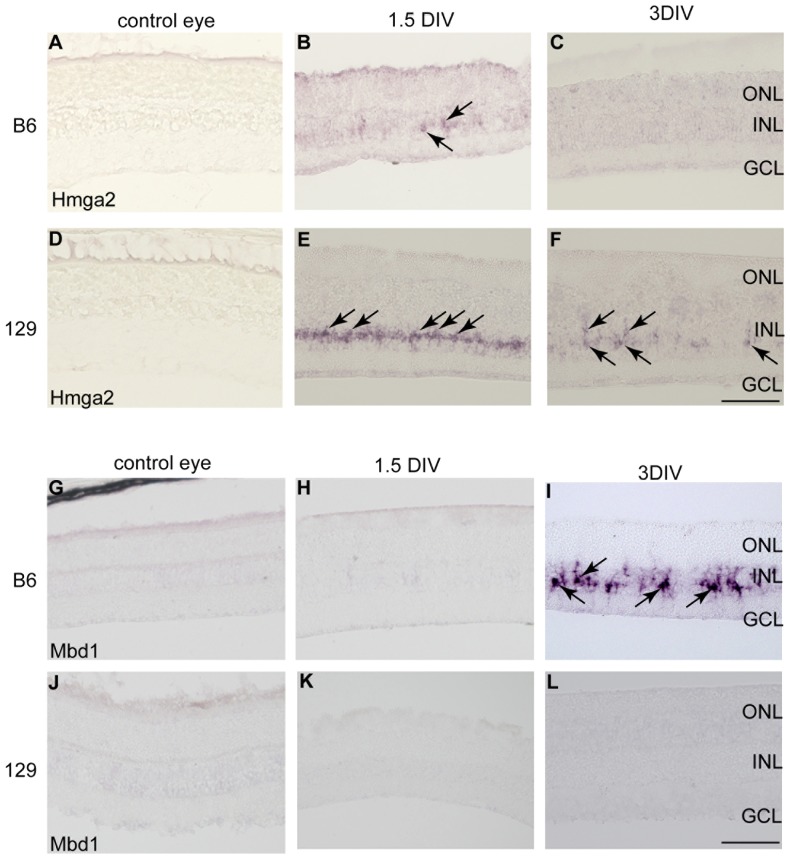
Temporal expression of *Hmga2* and *Mbd1* transcripts in the retinal explants from B6 and 129. (A, D) Hmga2 was not detected in the normal retina of B6 and 129. (B, E) Hmga2 was detected in the INL of the retinal explants at 1.5 DIV from both B6 and 129 (arrows). (C, F) Hmga2 was maintained only in the INL of the mouse retinal explant from 129 at 3DIV (arrows). (G–L) Mbd1 was not detected in the normal retina of B6 and 129 (G, J) or in the retinal explant at 1.5 DIV (H, K), but it was detected at 3DIV in the INL of B6 retinal explants (arrows) (I). (L) Mbd1 expression was not detected in the retinal explant from 129. Scale bar: 100 µm.

To detect genes whose expression was associated with the promotion or suppression of proliferation of Müller glia, we further selected 165 probes (126 genes) as candidates for proliferation repressors from group (a); these probes were based on the criteria that the gene expression difference was more than double between the two strains, while the basal expression was less than double between the two strains. Furthermore, the expression was more than double after making the explant culture in the B6 mouse retina (Fig. 5C, upper panel, Table 2). Conversely, we also selected 95 probes (78 genes) as candidate proliferation promoters from group (f) (Fig. 5C, lower panel, table 3). To examine which gene expression was likely to reflect the difference of the proliferative Müller glial response after damage, we compared the list of genes with previously reported genes selectively expressed in resting Müller glia [27], reactive Müller glia [28], [29], and retinal progenitor cells [30], [31]. We also checked the genes reportedly expressed in macrophage/microglia [32] because the above functional analyses suggested a difference in the innate immune responses in the retinal explants between B6 and 129. A relatively small number of genes enriched in normal Müller glia or in reactive Müller glia were included in the candidate proliferation repressors (7 of 126 genes: *fxyd3*, *Car6*, *GFAP*, *Cp*, *S100a6*, *A2m*, and *Bcl2a1*) and in the candidate proliferation promoters (4 of 78 genes: *Cxcl1*, *Pak3*, *Slc1a3*, and *Ednrb*). We considered a possibility that the basal expression levels of previously reported resting/reactive Müller glial marker genes were more than double between B6 and 129 and were excluded by our criteria. Only *Lcn2* was significantly highly expressed in B6 (×3.12), and only three genes (*Lgals3*; ×3.82, *Ednrb*; ×2.21, and *Serpina3n*; ×2.08) were highly expressed in 129. Therefore, it was likely that most of the previously reported genes selectively expressed in normal or reactive Müller glia changed their expression in a similar way in both B6 and 129 after damage. In consistent with the difference of Müller glial proliferation, the candidate proliferation promoter included retinal progenitor markers (*Hmga2*, *Ccnd1*, *Rrm2*, *Ltbp2*, *Fignl1*, and *Myc*). In contrast, the candidate proliferation repressor included only two genes, including *Car6*, which was expressed in both the retinal progenitor and normal Müller glia [33], and *Cp*, which was expressed in the reactive Müller glia. As suggested by the gene functional analyses, 14 genes in the candidate proliferation repressor were reported to be expressed in macrophage/microglia. In the candidate proliferation promoter, functional clustering analyses did not show a significant enrichment of a cluster related to the immune response; however, interferon inducible genes were relatively enriched (10 of 78 genes; *Ifi202b*, *H28*, *BC023105*, *Tgtp1*///*Tgtp2*, *Ifi203*, *Mx2*, *Gbp3*, *Gbp6*, *Iigp1*, and *Herc5*), and *Il6, Serpinb2*, *Tgtp1/Tgtp2*, *Mx2*, and *Gbp3* were expressed in microglia.

**Table 2:** Candidate proliferation repressors.

gene name	Affymetrix IDs	ratio to 129 in intact retina	ratio to 129 after damage	localization in retina
Gm12541	1443200_at	1.96	9.71	
Ang	1438937_x_at	1.50	8.93	
Ifi203///Ifi204///Ifi205///LOC640890///Mnda///Mndal	1452348_s_at	1.14	8.32	
Trim12	1437432_a_at	1.53	8.00	
Ifi205///Mnda	1452349_x_at	1.12	7.22	
Mbd1	1453678_at	1.21	6.69	reactive Müller glia (this research)
Gfap	1440142_s_at	1.88	4.91	reactive Müller glia [28]
---	1431225_at	1.70	4.60	
---	1458543_at	1.05	4.54	
Fst	1434458_at	0.78	4.12	
---	1436061_at	1.43	4.08	
Mbd1	1430838_x_at	0.98	3.97	
Ang	1438936_s_at	1.50	3.93	
---	1446661_at	0.91	3.88	
---	1420310_at	1.43	3.87	
S100a4	1424542_at	0.68	3.82	astrocyte [57]
Lyz2	1423547_at	1.50	3.76	
Arg1	1419549_at	1.08	3.73	
Gabrg3	1439717_at	1.68	3.72	
Ms4a6b	1418826_at	1.26	3.72	
Fxyd3	1418374_at	1.12	3.66	resting Müller glia [27]
2900057B20Rik	1431631_at	1.25	3.63	
---	1447329_at	1.07	3.46	
AA467197	1434046_at	1.91	3.40	
AI451617	1435665_at	0.80	3.31	
---	1433282_at	0.97	3.24	
4930579C15Rik	1453265_at	1.54	3.23	
Ctla2b	1452352_at	1.69	3.22	
Slc6a2	1460129_at	1.02	3.18	
Samd4	1436356_at	1.99	3.14	
Cd48	1427301_at	1.40	3.11	
Trim30	1417961_a_at	0.97	3.09	
E2f6	1437914_at	1.75	3.09	
9330161A08Rik	1459187_at	1.70	3.06	
Col4a6	1421007_at	1.49	3.05	
Ugt1a1///Ugt1a10///Ugt1a2///Ugt1a5///Ugt1a6a///Ugt1a6b///Ugt1a7c///Ugt1a9	1426261_s_at	1.23	3.01	
Ifi204	1419603_at	1.03	2.98	
Cd84	1422875_at	1.15	2.96	
Ccl9	1448898_at	1.27	2.95	macrophage/microglia [58]
Aif1	1418204_s_at	1.55	2.93	microglia [59]
Gm11428	1436530_at	1.15	2.89	
Ccl9	1417936_at	1.27	2.88	
Cp	1417497_at	1.20	2.87	reactive Müller glia, astrocyte [29], retinal progenitor [30]
Stfa2l1	1442339_at	1.03	2.86	
8430419K02Rik	1460515_at	1.31	2.85	
6720422M22Rik	1437798_at	1.63	2.85	
Wdr20a	1428039_at	1.79	2.85	
Fam101a	1453192_at	1.61	2.82	
Ly86	1422903_at	1.73	2.82	macrophage/microglia [60]
Cp	1448735_at	1.27	2.81	reactive Müller glia, astrocyte [29], retinal progenitor [30]
---	1445944_at	1.86	2.76	
Ifi27l2a	1426278_at	1.14	2.73	
Clec7a	1420699_at	1.25	2.73	macrophage/microglia [61]
Fcrls	1448891_at	1.08	2.71	
Fxyd1	1421374_a_at	1.26	2.70	
Mbd1	1417968_a_at	1.07	2.70	
Pnma2	1437018_at	1.69	2.68	
2410018L13Rik	1431317_at	1.84	2.67	
---	1444607_at	0.86	2.64	
Pgf	1418471_at	1.29	2.61	
Lin7b	1439239_at	1.32	2.61	
---	1442947_x_at	0.96	2.60	
Car6	1421001_a_at	0.95	2.60	retinal progenitor, Müller glia [33]
Kctd14	1426633_s_at	1.90	2.60	
Bcl3	1418133_at	1.31	2.58	
Mbd1	1430837_a_at	1.11	2.58	
Plscr2	1448961_at	1.48	2.58	
Raly	1430465_at	1.82	2.55	
---	1447081_at	1.12	2.54	
Adamts4	1455965_at	1.28	2.52	
Espn	1423005_a_at	1.36	2.52	Photoreceptor [62]
Ccl12	1419282_at	1.55	2.51	Microglia [63]
Tnfaip2	1438855_x_at	1.07	2.51	
Parp3	1451969_s_at	1.14	2.50	
S100a6	1421375_a_at	0.92	2.46	reactive Müller glia [28]
Fam132a	1439422_a_at	1.78	2.46	
Pycard	1417346_at	1.18	2.43	
C87414	1455830_s_at	1.03	2.43	
Rab6b	1460617_s_at	1.57	2.42	
2900017F05Rik	1430096_at	1.74	2.41	
March6	1445928_at	1.85	2.39	
2810047C21Rik1///Gm3912	1453241_a_at	1.46	2.39	
Ephx1	1422438_at	1.33	2.39	
Ms4a6d	1419599_s_at	1.28	2.39	
Fcgr1	1417876_at	1.08	2.38	Microglia [32]
---	1441389_at	1.37	2.38	
Cited4	1425400_a_at	1.39	2.37	
AI451617///Trim30	1456494_a_at	1.03	2.36	
C1qc	1449401_at	1.84	2.35	
Ptgs1	1436448_a_at	1.17	2.35	retinal neuron, microglia [64]
Afap1l2	1436870_s_at	1.18	2.33	
2700079J08Rik///Ccrn4l///Gm4638	1436362_x_at	1.96	2.32	
Mlc1	1448139_at	1.06	2.32	retinal ganglion cell [65]
Lin7b	1449172_a_at	1.35	2.31	
Lgals9	1421217_a_at	1.22	2.31	
Bmp1	1427457_a_at	1.19	2.31	
9430065F17Rik	1432320_at	1.72	2.31	
---	1447288_at	1.00	2.31	
---	1446042_at	1.15	2.30	
A2m///LOC677369	1434719_at	1.80	2.28	Muller glia [66]
Lin7b	1439240_x_at	1.08	2.28	
Mgp	1448416_at	1.69	2.28	retinal ganglion cell [67]
Zfp30	1446313_at	1.42	2.27	
Gstm1	1416416_x_at	1.75	2.27	
Trub1	1428281_at	1.97	2.27	
Ctss	1448591_at	1.47	2.26	
Serping1	1416625_at	1.66	2.26	
Ccl6	1420249_s_at	0.79	2.24	macrophage/microglia [68]
Gstm1	1448330_at	1.71	2.24	
---	1458589_at	1.24	2.24	
Ms4a6d	1419598_at	0.94	2.23	
---	1458382_a_at	1.07	2.23	
Fam46c	1448021_at	1.04	2.22	
C4b	1418021_at	1.59	2.21	
Cyb561	1417507_at	1.93	2.21	
---	1441932_at	1.39	2.20	
Tyrobp	1450792_at	1.73	2.19	Microglia [69]
---	1442862_at	1.23	2.19	
Pisd-ps1///Pisd-ps2///Pisd-ps3	1454566_at	1.73	2.19	
Lin7b	1418683_at	1.24	2.18	
D630033O11Rik	1443458_at	0.95	2.18	
C5ar1	1439902_at	0.93	2.18	retinal neuron and microglia [70]
Mndal	1452231_x_at	1.82	2.18	
Apobec2	1417889_at	1.43	2.17	regenerating Muller glia [56]
Myo1g	1427892_at	1.28	2.16	
Fam173b	1428683_at	1.60	2.16	
4833408G04Rik	1454409_at	1.61	2.14	
---	1447123_at	0.84	2.14	
Bcl2a1a///Bcl2a1b///Bcl2a1d	1419004_s_at	1.49	2.13	reactive Muller glia [71]
Itgam	1422046_at	1.01	2.11	Microglia [32]
Crispld2	1434758_at	0.91	2.11	
Ccl6	1417266_at	1.18	2.10	
D4Ertd617e	1457668_x_at	1.88	2.09	
Oxct1	1436750_a_at	1.40	2.07	
Zdhhc14	1438975_x_at	1.64	2.07	
---	1444620_at	1.60	2.07	
Ms4a6c	1450234_at	1.23	2.07	
Hebp2	1449271_a_at	1.37	2.06	
Pisd-ps1///Pisd-ps3	1435353_a_at	1.24	2.06	
Bik	1420362_a_at	1.39	2.06	
Shisa3	1460000_at	1.09	2.06	
Asf1b	1423714_at	1.34	2.06	
Kcng4	1428536_at	1.11	2.05	bipolar cell [72]
Pkig	1423945_a_at	1.92	2.05	
Mxra7	1453855_at	1.69	2.05	
---	1446017_at	0.76	2.05	
Itgb2	1450678_at	1.42	2.05	Microglia [73]
Timp1	1460227_at	0.70	2.04	Astrocyte [74]
Trem2	1421792_s_at	1.50	2.04	Microglia [75]
Fam132a	1417393_a_at	1.67	2.04	
C3ar1	1442082_at	1.01	2.04	Microglia [32]
Lin28a	1437752_at	1.53	2.04	regenerating Muller glia [9]
Ube2l6	1417172_at	1.25	2.03	
Casp1	1449265_at	0.95	2.02	
Gvin1	1429184_at	1.85	2.02	
Fam132a	1448687_at	1.50	2.02	
Fgf2	1449826_a_at	1.03	2.02	Photoreceptor [29]
---	1438027_at	1.55	2.01	
Oxct1	1455804_x_at	1.34	2.01	
---	1443777_at	0.97	2.01	
Pmaip1	1418203_at	0.95	2.01	
1110008P14Rik	1423947_at	1.37	2.00	
Cxcl16	1449195_s_at	0.88	2.00	resting Muller glia [27]
Ang	1450717_at	1.19	2.00	

**Table 3:** Candidate proliferation promoters.

gene name	Affymetrix IDs	ratio to B6 in intact retina	ratio to B6 after damage	localization in retina
Ifi202b	1457666_s_at	1.21	111.82	
Ifi202b	1421551_s_at	0.97	38.20	
H28	1425917_at	1.18	11.51	
H28	1421596_s_at	1.04	7.20	
1810011O10Rik	1451415_at	1.78	6.85	
Hmga2	1422851_at	0.88	6.67	reactive Müller glia (this research), developing retinal margin [34]
Hmga2	1450780_s_at	0.65	5.80	
Hmga2	1450781_at	1.01	5.74	
H2-T24	1422160_at	0.48	5.30	
Opn4	1445121_at	1.04	4.93	retinal ganglion cell [76]
BC023105	1425394_at	0.89	4.31	
Matn2	1419442_at	1.50	4.29	
Matn2	1455978_a_at	1.49	3.89	
Ddx60	1439114_at	1.02	3.60	
Tgtp1///Tgtp2	1449009_at	0.93	3.56	macrophage/microglia [77]
Rnd3	1416701_at	1.45	3.54	
Npvf	1421686_at	1.59	3.48	
Ednrb	1423594_a_at	1.87	3.47	reactive Müller glia, astrocyte [29]
Ifi203	1451567_a_at	0.96	3.35	
Pde2a	1452202_at	0.98	3.32	
Serpinb2	1419082_at	0.82	3.23	macrophage/microglia [78]
Notch4	1449146_at	0.96	3.15	reactive Müller glia (this research)
Ldb3	1433783_at	0.66	3.13	
Gm3756///Gm5620///Gm6682///Gm7172///LOC100044416///LOC100045728///Tuba1a///Tuba1b///Tuba1c	1448232_x_at	0.93	3.03	regenerating Müller glia [14]
Ets1	1452163_at	1.14	3.01	
Mx2	1419676_at	0.94	2.88	macrophage/microglia [79]
Trim34	1426093_at	1.00	2.88	
Gbp3	1418392_a_at	0.51	2.88	macrophage/microglia [80]
Gbp6	1425156_at	0.95	2.85	
Cxcl1	1419209_at	1.18	2.84	resting Müller glia [27]
BC006779	1435454_a_at	1.21	2.83	
Gbp6	1434380_at	0.86	2.83	
Tubb6	1416431_at	1.11	2.80	
Anxa4	1424176_a_at	1.57	2.69	
Il6	1450297_at	0.92	2.66	Microglia [32]
Uhrf1	1415810_at	0.68	2.65	
Pak3	1417924_at	1.50	2.64	resting Müller glia [27], photoreceptor [81]
Ifi47	1417292_at	1.09	2.62	
---	1436633_at	1.40	2.62	
Pde2a	1447707_s_at	1.05	2.56	
Ccnd1	1417419_at	0.89	2.55	retinal progenitor [30]
Ets1	1426725_s_at	1.30	2.51	
Hells	1417541_at	1.12	2.48	
Gm3417///Gm3448///Tcte3	1421682_a_at	1.66	2.48	
Anxa4	1457658_x_at	1.29	2.47	
Rrm2	1448226_at	1.14	2.46	retinal progenitor [30]
Iigp1	1419043_a_at	0.77	2.44	
Fxyd2	1419378_a_at	1.27	2.44	
Iigp1	1419042_at	0.86	2.40	
Ndp	1449251_at	1.07	2.39	Müller glia [82]
Rnd3	1416700_at	1.56	2.35	
Dscc1	1452912_at	0.93	2.33	
Btc	1421161_at	1.02	2.31	
Serpine1	1419149_at	1.21	2.29	
Met	1434447_at	0.95	2.28	
Gm3756///Gm5620///Gm6682///Gm7172///LOC100044416///LOC100045728///Tuba1a///Tuba1b///Tuba1c	1416128_at	1.30	2.27	
Herc5	1438037_at	0.71	2.26	
3930401B19Rik///A130040M12Rik///E430024C06Rik	1453238_s_at	1.07	2.26	
Dtna	1419223_a_at	0.99	2.25	Müller glia [83]
Clcf1	1437270_a_at	1.28	2.24	
Upp1	1448562_at	0.88	2.23	
Tfdp2	1443962_at	1.18	2.23	
Nav3	1456144_at	1.05	2.23	
Cd14	1417268_at	1.24	2.21	
Msn	1450379_at	0.98	2.21	
Hmga1///Hmga1-rs1	1416184_s_at	0.84	2.19	
Rai14	1417400_at	1.61	2.19	Retinal ganglion cell, Müller glia[84]
Pbk	1448627_s_at	1.33	2.19	
Fignl1	1422430_at	0.94	2.18	retinal progenitor, resting Müller glia [85]
Krt24	1453327_at	1.12	2.18	
9230105E10Rik	1443858_at	1.14	2.17	
Slc1a3	1426341_at	1.43	2.17	resting Müller glia [27], retinal progenitor [30]
Ddx60	1451777_at	1.01	2.16	
Rrm2	1434437_x_at	0.83	2.14	retinal progenitor [30]
Oaf	1424086_at	0.87	2.14	
Tubb2a-ps2///Tubb2b	1449682_s_at	1.08	2.14	
Ltbp1	1448870_at	0.74	2.13	developing retinal margin [31]
Kitl	1448117_at	1.35	2.13	retinal ganglion cell [86]
Sfrs3	1438215_at	1.42	2.12	
Ednrb	1426314_at	1.13	2.12	reactive Müller glia [29]
Fosl1	1417487_at	0.77	2.12	
Pml	1456103_at	0.85	2.12	
Eif4ebp1	1434976_x_at	0.88	2.09	
LOC100047360///Scml2	1456984_at	1.07	2.09	
Rsad2	1421008_at	1.14	2.08	
Lactb2	1425140_at	1.38	2.07	
Thbd	1448529_at	0.78	2.07	
Nusap1	1416309_at	1.05	2.06	
Inpp5f	1442100_at	1.39	2.06	
Akr1b8	1448894_at	0.67	2.05	
Il1rap	1449585_at	1.04	2.05	
---	1445381_at	0.92	2.03	
Pml	1459137_at	0.93	2.02	
Plat	1415806_at	1.54	2.02	
Myc	1424942_a_at	0.88	2.01	developing retinal margin [31]

### Chromatin remodeling factors were differentially expressed in B6 and 129

When ordered according to the ratio of the expression levels between B6 and 129 at 3DIV, *Hmga2* (high mobility group AT-hook 2) was fourth among the proliferation promoters (×6.67, ×5.8, and ×5.74 for each probe) (Table 3). Hmga2 is a chromatin remodeling factor, which is widely expressed and includes retinal progenitors during embryogenesis [34], but is not detected in adult retina. Recently, Hmga2 was reported to promote neural stem cell self-renewal in the embryonic and early postnatal mouse brain [35] and to regulate the neurogenic potential characteristics of early-stage neural precursor cells [36]. Interestingly, we found another chromatin remodeling factor, *Mbd1* (methyl-CpG binding domain protein 1), among the candidate proliferation inhibitors (Table 2). In contrast to Hmga2, Mbd1 was reported to negatively regulate the proliferation of adult neural stem cells via the repression of *Fgf2* and *mir-184* transcription [37], [38]. To evaluate the localization of Hmga2 and Mbd1 mRNA in the retinal explant, we performed *in situ* hybridization. In the control adult normal retina, Hmga2 was not detected in either B6 or 129. In the retinal explant from 129, Hmga2 was detected in the INL at 1.5 DIV. The Hmga2 transcript was maintained in a small number of cells in the INL at 3DIV. The Hmga2 transcript was detected around the nucleus and also in the processes extending to the ONL side, reminiscent of Müller glia. In contrast, in the retinal explant from B6, we detected a faint expression of Hmga2 at 1.5 DIV, but not at 3DIV. Mbd1 expression was also not detected in the control retina, but was upregulated in the INL of B6 mouse retinal explants at 3DIV. These data suggested that the cell cycle progression in Müller glia was associated with the expression of chromatin remodeling factors in 129 and B6.

## Discussion

The difference in the intrinsic potency of retinal regeneration across different species has been an issue of interest for researchers, particularly the fact that the mammalian retina has a rather lower potency of regeneration compared with some other vertebrates [11]. Nevertheless, it has been widely recognized that regenerative processes do occur in the mammalian brain [39], and identifying what regulates a permissive or an inhibitory environment for Müller glia proliferation in the mammalian retina may provide a new clinical therapeutic strategy. By comparing mouse strains with high or low Müller glia proliferative potency in the present study, we gained insight into the differences in the regenerative environments in these mouse strains. We observed significantly more Müller glia proliferation in the retinal explants from 129 and BDF1 compared with B6. The proliferation of Müller glia in 129 and BDF1 was also responsive to a GSK3 inhibitor. Müller glia in both 129 and B6 showed the expression of retinal progenitor markers and a transient increase in Cyclin D1, but the cell cycle progress indicated by Ki67 and the incorporation of EdU was only observed in 129; these findings imply that Müller glia were similarly de-differentiated and re-entered the cell cycle, but in B6 retinas, the cells were trapped in the G1 phase. We further showed the gene expression profiles that may support the different responses between B6 and 129 after retinal damage.

### The degree of Müller glial proliferation varies between mouse strains

In previous reports, B6 and 129 mouse strains had a significant difference in the neurogenesis of the adult hippocampus [40]. The effects of genetic differences on the proliferation of adult neural stem cells were not uniform across environmental conditions. In the normal condition, adult neural stem cells in the mouse dentate gyrus showed a significantly higher proliferation ratio in B6 compared with 129 [40]. However, when mice were put in an enriched environment, a significant increase in BrdU-positive cells in the dentate gyrus was observed in 129, whereas no change in proliferation was detected in B6 [41]. Müller cells in the retina share many common features with radial glia in the brain [5]. Thus, in damaged retina, a shift of Müller glia from a quiescent to a proliferative state might be regulated in a similar way to the increase in neural stem cell proliferation in the stimulated brain, and we previously showed that Müller glia may contribute to regeneration in rat models [1], [2]. In the current study, we also showed a consistent phenomenon in mice, although the Müller glial response differed across strains. Our retinal explant culture, which caused preferential photoreceptor cell death, evoked a significantly larger proliferation of Müller glia in 129 compared with B6. Differences in the number of proliferative Müller glia were further distinguished when a GSK3 inhibitor was added in the culture medium: the number of EdU-positive Müller glia significantly increased in the retinal explants from 129, BDF1, Balb/c, and ICR, but very few EdU-positive Müller glia were detected in the retinal explants from B6 (Fig. 1 H, and data not shown). We also tested other reported mitogens for Müller glia after retinal damage, such as Wnt3a [2], EGF [3], and a combination of insulin and FGF [3]. Similar to the GSK3 inhibitor, these factors increased the number of BrdU-positive Müller glia in the retinal explants from 129, but not in that from B6 (data not shown). Because canonical Wnt signaling, EGF signaling, and FGF signaling were mediated by a GSK3 protein [87], [88], it was not clear which mitogenic signals mainly contributed to the difference in the degree of Müller glial proliferation between the mouse strains. In contrast to zebrafish, in which HB-EGF induces proliferation of Müller glia in the undamaged retina [10], Müller glia did not proliferate when a Wnt protein, an EGF protein, or a GSK3 inhibitor was added to the undamaged rodent retina [3] (Osakada, unpublished data). In conjunction with our results that Müller glia in B6 poorly responded to a number of mitogens, the damage signals that were required for Müller glial proliferation might be suppressed in B6, unlike in other mouse strains.

### Cyclin D1 expression level was associated with Müller glial proliferation

We detected a lower expression of Cyclin D1 in the retinal explant from B6 compared with that from 129. Cyclin D1 is expressed in the proliferating retinal progenitors, and the loss of Cyclin D1 causes hypocellular retinas due to impaired retinal progenitor proliferation and photoreceptor cell death [21], [22], [42]. Conversely, the overexpression of Cyclin D1 accelerates cell cycle progression according to the expression levels in rat fibroblasts [43]. The role of Cyclin D1 in the retinal progenitor is not simple, and part of its role seemed to change dependent on the developmental stage. Cyclin D1 deficient retinal progenitors showed an elongated cell cycle and prematurely exited the cell cycle in the embryonic retina [44]. On the other hand, in the postnatal retina, Cyclin D1 deficient proliferating retinal progenitors also had an elongated cell cycle, but were maintained in the central part of the retina where histogenesis was already terminated in the wild type retina [45]. Thus, the role of Cyclin D1 for cell cycle exit seemed to be different between the embryonic and the postnatal retinal progenitors. However, in both periods, Cyclin D1 was required to keep the cell cycle length short. Therefore, it was possible that in the B6 retina, the accumulation of Cyclin D1 was not sufficient to induce proliferation of Müller glia within 4 days of culture. We did not confirm whether the Cyclin D1 expression level further increased after 4DIV. However, because the increase in proliferative Müller glia was not detected at 7DIV in B6, it was not likely that the Müller glia in the B6 retina began to proliferate later than those in the 129 retina after damage. The factors that caused the difference in Cyclin D1 expression levels between B6 and 129 after damage should be investigated in a future study. Multiple signals are known to regulate Cyclin D1 expression [46], and the sustained activation of the MAP kinase/ERK pathway is required for the continuous expression of Cyclin D1 during the G1 phase [47]. Together, our gene expression profile showed a higher expression of genes for the “positive regulators for MAP kinase activity” in 129 after retinal damage, implying that the differences in the regulation of the MAP kinase pathway may contribute to the difference in the number of proliferative Müller glia via the regulation of Cyclin D1 expression.

### Identification of new Müller glial markers differentially expressed in B6 and 129 after retinal damage

Our gene profiling analysis also suggested several factors associated with the suppression or promotion of Müller glia proliferation after retinal damage.

Only a small number of previously reported genes enriched in resting and reactive Müller glia were differentially expressed between B6 and 129 after retinal damage. Our gene expression analyses from normal and damaged retina suggested that the average expression levels of Müller glial marker genes were similar between B6 and 129 in normal and damaged retina. Because proliferative Müller glia were only a small population of retinal Müller glia even in 129, it might be difficult to detect if some reactive Müller glial marker genes changed their expression levels only in these small populations. One additional possibility was that despite the entry into the cell cycle, the proliferative Müller glia maintained their characteristic Müller glial gene expression similar to the non-proliferative glia. Because we detected EdU positive Müller glia also expressed in the GS, it was not likely that the proliferative Müller glia completely lost their mature gene expression during the cell cycle.

On the other hand, we found that two chromatin remodeling factors, *Hmga2* and *Mbd1*, were distinctly expressed in the retinal explant from 129 and B6. Hmga2 is a chromatin-associated protein that can modulate transcription by altering the chromatin architecture [54]; it also has roles in maintaining the young neural stem cell state in development [35], [36]. We observed an upregulation of Hmga2 mRNA in Müller glia at 1.5DIV in both B6 and 129 mouse retinal explants and a sustained expression in a portion of Müller glia at 3DIV only in 129. The Hmga2 mRNA is degraded by let-7 miRNA in several cancer cell lines [54], [55], and the loss of let-7 induces the expression of Hmga2. Interestingly, a cascade of Ascl1-Lin28 and a downregulation of let-7 regulate the de-differentiation and proliferation of Müller glia in the zebrafish retina after damage [9]. We further examined whether the reported upstream factors for let-7 miRNA were differentially expressed between B6 and 129. Curiously, *Lin28a* expression was higher in the non-proliferative B6 retina (Table 2). Because *Lin28* was rapidly induced in 15 hours after retinal damage in the zebrafish retina, we examined the expression levels of *Lin28a* and *Lin28b* at 1DIV and 3DIV by quantitative RT-PCR (data not shown). *Lin28a* expression levels increased from the normal retina to 1DIV in both B6 and 129, without a significant difference between the two strains. Then, *Lin28a* expression levels decreased at 3DIV, and the relative expression level was significantly higher in B6 (×1.98). On the other hand, *Lin28b* expression levels showed a trend to increase only in 129 at 1DIV, but decreased at 3DIV. However, *Lin28b* expression levels were not significantly different between B6 and 129 at any time points. We also examined the expression levels of a further upstream factor, *Ascl1a*, but did not detect a significant increase at 8 hr, 1DIV, 2DIV, or 3DIV in B6 and 129. Thus, different from the robust and continuous upregulation of Lin28 in the zebrafish retina after injury [9], the *Lin28a/b* expression level mildly and only transiently increased after a retinal explant in the mouse. Because the increase in Lin28 was much milder and shorter in the optic nerve crash model of the zebrafish [56], it was still possible that the relatively mild upregulation of *Lin28* in the mouse retinal explant contributes to the Müller glial proliferation. Further functional analysis would clarify if the same molecular cascade regulates the de-differentiation and proliferation of Müller glia in the zebrafish and the mammalian retinas.

### Different retinal environments may affect Müller glial proliferation after damage

Our microarray data suggested that genes related to the inflammatory response were significantly enriched in the B6 retina after damage. On the other hand, interferon-inducible genes were relatively enriched in 129 after damage. In the retinal tissue, the secretion of inflammatory cytokines and interferon were both from activated microglia through TLR signaling [32]. The induction of inflammatory cytokines was generally mediated by NF-kappaB, whereas the induction of interferon was mediated by IRF3 [48]. Although it is still not clear whether inflammation is detrimental or beneficial for the regeneration of central nervous system tissue, evidence suggests that the activation of microglia by an acute injury suppresses the proliferation of neural progenitor/stem cells in the brain [49], [50], [51]. In the retina, activated microglia also inhibited the proliferation of Müller glia [52] *in vitro*. We detected Ki67-positive and Iba-1 positive proliferating microglia in the ONL of retinal explants. Although the proliferating microglia were detected in both B6 and 129, different responses of microglia may affect the number of proliferative Müller glia.

One other candidate factor contributing to the proliferation-repressive environment in the B6 mouse retina was Edn2 signaling. Among the candidate proliferation repressors, *Gm12541*, a non-coding RNA overlapping the coding region of *Fgf2* and its 5′ end, was most differentially highly expressed. Recently, *Gm12541* and *FGF2* were shown to be induced in the rhodopsin mutant retina dependent on *Edn2* [53]. We also found that several candidate mediators of the neuroprotective effects of Edn2 [53] were involved in the candidate proliferation repressors (*Gm12541*, *Gfap*, *Fgf2*, *Serping1*, *S100a6*, *C4b*, and *A2m*). *Edn2* was shown to be expressed in photoreceptors after light-induced photoreceptor damage and in diverse mouse models of photoreceptor degeneration [29]; it was also upregulated in our retinal explant culture in both B6 and 129 (data not shown). Interestingly, *Edn2* expression was significantly higher in B6 in the normal retina (×7.37), but its receptor *Ednrb* was expressed higher in 129 after damage (×3.47 and ×2.11). It was shown that when *Edn2* was induced by preconditioning with mild-intensity light, its receptor *Ednrb* was not induced even after strong light stimulation [29]. Considering a neuroprotective effect of Edn2 through Fgf2 [53], the higher expression of Edn2 in the normal B6 retina may contribute to the neuroprotective environment after damage. In addition, the genes expressed in the photoreceptor, which have important roles for visual functions, were preferentially downregulated in 129 after damage (group (b)). Considering that the extent of photoreceptor degeneration is one key to induce Müller glia-derived regeneration in zebrafish [15], the neuroprotective environment in the B6 retina after damage might prevent Müller glial proliferation. It should be further elucidated whether an Ednrb mediated signal promotes Müller glial proliferation, and, if so, how the Edn2/Ednrb signal is segregated to neuroprotective and proliferation-inducing signals after damage.

In summary, we compared two mouse strains, 129 and B6, with high and low potencies of injury-induced Müller glia proliferation. In B6, the inhibitory environment for Müller proliferation in the retinal explant was associated with lower expression levels of Cyclin D1 and Nestin compared with those in 129, which seemingly resulted in cell cycle arrest at the G1 phase in B6 Müller cells. We also found that the expression of genes related to the immune response was increased in B6, and two chromatin remodeling factors that had distinct roles in the maintenance of neural stem cells were differentially expressed in the retinal explants from B6 and 129 mice. These findings are consistent with the idea of an inhibitory environment for regeneration in the B6 strain.
